# Novel mass spectrometry based detection and identification of variants of rabies virus nucleoprotein in infected brain tissues

**DOI:** 10.1371/journal.pntd.0006984

**Published:** 2018-12-14

**Authors:** Matthew Reed, Olga Stuchlik, William C. Carson, Lillian Orciari, Pamela A. Yager, Victoria Olson, Yu Li, Xianfu Wu, Jan Pohl, Panayampalli Subbian Satheshkumar

**Affiliations:** 1 Biotechnology Core Facility Branch, Centers for Disease Control and Prevention, Atlanta, GA, United States of America; 2 Poxvirus and Rabies Branch, Centers for Disease Control and Prevention, Atlanta, GA, United States of America; Wistar Institute, UNITED STATES

## Abstract

Human rabies is an encephalitic disease transmitted by animals infected with lyssaviruses. The most common lyssavirus that causes human infection is rabies virus (RABV), the prototypic member of the genus. The incubation period of RABV in humans varies from few weeks to several months in some instances. During this prodromal period, neither antibodies nor virus is detected. Antibodies, antigen and nucleic acids are detectable only after the onset of encephalitic symptoms, at which point the outcome of the disease is nearly 100% fatal. Hence, the primary intervention for human RABV exposure and subsequent post-exposure prophylaxis relies on testing animals suspected of having rabies. The most widely used diagnostic tests in animals focus on antigen detection, RABV-encoded nucleoprotein (N protein) in brain tissues. N protein accumulates in the cytoplasm of infected cells as large and granular inclusions, which are visualized in infected brain tissues by immuno-microscopy using anti-N protein antibodies. In this study, we explored a mass spectrometry (MS) based method for N protein detection without the need for any specific antibody reagents or microscopy. The MS-based method described here is unbiased, label-free, requires no amplification and determines any previously sequenced N protein available in the database. The results demonstrate the ability of MS/MS based method for N protein detection and amino acid sequence determination in animal diagnostic samples to obtain RABV variant information. This study demonstrates a potential for future developments of rabies diagnostic tests based on MS platforms.

## Author summary

Although rabies is almost always fatal after the symptom onset phase, it can be prevented by timely administration of post-exposure prophylaxis (PEP), which involves passive antibody transfer and vaccination. One of the primary laboratory confirmatory tests for RABV infection is antigen detection, directed against the RABV encoded N protein using anti-N protein specific antibodies, in central nervous system (CNS) tissue samples of animals. This immuno-microscopy based detection utilizes either fluorescent tags (direct detection) or chromogenic substrates (indirect) in brain impressions from animals in which rabies is suspected. In this study, we explored the detection of N protein by a novel mass spectrometry (MS) based method that is label-free and does not require target amplification. The MS method specifically detected N protein in brain tissue and identified RABV variants based on amino acid sequence information. To our knowledge, this is the first report of an N protein detection method that does not utilize either antibodies or microscopy. This method provides an alternative platform for the development of future rabies diagnostic tests.

## Introduction

Mass spectrometry (MS) based proteomics is a collection of rapidly evolving techniques being utilized as a diagnostic tool for infectious diseases. MALDI-TOF MS (matrix-assisted laser desorption/ionization–time-of-flight) is being used in clinical microbiology laboratories to identify an impressive spectrum of bacteria and fungi from infected specimens (generally enriched by culturing prior to detection) [1, 2]. At present, there is no comparable MS-based approach for identification of viruses. As the majority of viruses encode only a few proteins, most of the proteome in diagnostic samples like plasma or tissue samples will be dominated by the host [3]. However, if viral proteins accumulate at high concentration or there was shutdown of host protein synthesis, it might be feasible for direct detection of viral proteins from diagnostic samples. As the primary diagnosis in post-mortem animals for rabies relies on viral protein detection in infected samples, we explored MS as a diagnostic option. In this study, we explored LC-ESI-MS/MS (liquid chromatography–electrospray ionization tandem mass spectrometry) for peptide mass and amino acid sequence determination.

Rabies is an ancient disease known to humanity for over 5000 years. Initially, clinical signs exhibited by animals such as excessive salivation, aggression, choking or gagging were used to diagnose if the animal was rabid [4]. Once the causative agent of the disease was determined to be a viral infection, diagnosis relied on the ability of brain homogenate from suspect animal to cause infection in naïve animals or cell cultures [5–8]. Both methods required several days to weeks to confirm a RABV infection. The major breakthrough in rabies diagnosis was achieved after the invention of microscopic methods and histostaining, although initial methods still depended on non-specific staining reagents like Sellers stain to identify and differentiate Negri bodies, intra-cytoplasmic inclusions in the brain tissue of infected animals [9]. The inclusions were later characterized as a ribonucleoprotein complex (RNP), predominantly comprised of nucleoprotein (N protein) expressed from the viral genome. RABV, the causative agent of rabies, is a bullet-shaped virus belonging to the Lyssavirus genus. Lyssaviruses are in the order *Mononegavirales* and the family *Rhabdoviridae*, characterized by a 12 kilobase unsegmented negative-sense RNA genome. The genome encodes five proteins starting with the N protein closest to the 3’ end, followed by the phosphoprotein (P protein), the matrix protein (M protein), the glycoprotein (G protein) and the large RNA dependent RNA polymerase (L protein) [10]. These proteins are differentially expressed, the genes closer to the 5’ end of positive sense RNA (or 3’ end of negative sense genome) are expressed at higher levels compared to downstream genes (farther from 5’ end). The N protein mRNA transcripts are transcribed at higher levels to make it the most abundant viral protein synthesized after RABV infection [11]. N protein along with P and L proteins coat the viral genome to form the RNP, which accumulates in the cytoplasm of infected cells as large or granular inclusions [12].

Once immunological methods were developed, antibodies generated against RABV or RNP, comprised predominantly against the N protein, were utilized for specific detection of RABV antigens (proteins expressed from RABV genome). The most widely used rabies diagnostic method, the direct fluorescent antibody (DFA) test or fluorescent antibody test, detects the N protein in brain tissue impressions with polyclonal or monoclonal antibodies (mAbs) directly conjugated to fluorescent compounds [13–15]. The DFA test is considered the gold standard in rabies diagnostics for the detection of RABV antigen in animal brain tissues suspected of rabies [15, 16]. Alternatively, modified chromogenic-based detection methods, such as the direct rapid immunohistochemistry test (DRIT), have also been developed for rabies diagnosis using anti-N mAbs or polyclonal antibodies without the need for fluorescent microscopy [17, 18]. Non-microscopy based protein detection techniques for rapid, point of care diagnostics, such as the rabies immunochromatographic diagnostic (RID) tests, commonly known as lateral flow assay (LFA) are available. LFAs have provided mixed results with concerns on specificity and sensitivity of N protein detection [19–21]. The assay still relies on using a combination of antibodies specific to N protein and the ability to capture and detect protein in infected tissue lysates, which can be visualized by a colored band on LFA strips. Currently, these tests are not yet approved for regular diagnostics by the World Health Organization, but are helpful in countries where surveillance is lacking and for epidemiological studies [19].

In this study, we explored MS as an alternative method for rabies N protein detection. MS is an unbiased proteomics approach, non-amplifying, non-sequence specific technique and does not require specific reagents for protein detection [3, 22]. We demonstrate detection of all RABV encoded proteins in purified or crude infected cell lysates by MS. Additionally, N protein was detected and the amino acid sequence was determined by MS/MS-based peptide fragment mass information from animal diagnostic samples for several RABV variants circulating in the United States (U.S.).

## Methods

### Cells and viruses

BSR (a clone of Baby Hamster Kidney 21 cells) or mouse neuroblastoma (MNA) cells (CDC collection) were cultured in E-MEM supplemented with 10% FBS (Fetal bovine serum) containing antibiotics (Penicillin and Streptomycin) and antimycotic (Amphotericin B) essential vitamins and L-glutamine. BSR cells were infected with the RABV ERA (Evelyn Rokitnicki Abelseth) virus strain at 0.01 multiplicity of infection for 2–5 days. The media supernatant was harvested, subjected to low speed centrifugation, followed by sucrose density gradient centrifugation to purify ERA virus particles. MNA cells (T75 flask) were either mock-infected or infected with RABV CVS-11 (challenge virus strain, 10 X TC ID_50_) for 24 h, washed with PBS and harvested by centrifugation. The cell pellet was resuspended in 1X NuPAGE LDS sample buffer containing reducing agent (ThermoFisher).

### DFA test and antigenic typing

Brain samples from animals (CDC collection) were submitted by the state public health, state veterinary, and US Department of Agriculture rabies laboratories for confirmatory testing and antigenic typing. Acetone-fixed brain impressions were tested by the standard DFA using the pre-calculated optimal working dilutions of two FITC anti-rabies mAb conjugates (Millipore Sigma Light Diagnostics and Fujirebio Diagnostics) as per the National Standard Protocol for rabies diagnosis (https://www.cdc.gov/rabies/pdf/rabiesdfaspv2.pdf). The two mAbs cocktails have different epitope recognition and affinity/avidity differences are required for DFA confirmatory testing. Similarly, a non-rabies antibody FITC conjugate, negative control reagent (Millipore Sigma Light Diagnostics), which contains the same IgG isotypes as the rabies specific antibody, is used for specificity. All controls (positive and negative) demonstrated the expected results. Samples demonstrating the presence of rabies-specific antigen in brain impressions (typical 4+ sparkling apple-green fluorescent inclusions with both anti-rabies conjugates and no specific fluorescence demonstrated with the non-rabies conjugate) were reported as positive. Based on the level of N protein specific staining, the samples are classified as 1+ to 4+ distribution, where 4+ demonstrate maximum staining (https://www.cdc.gov/rabies/pdf/rabiesdfaspv2.pdf). While most samples have either 3+ or 4+ antigen levels, around 5% demonstrate lower levels of N protein staining by DFA. For validation with MS assay, we included samples from 1+ to 4+ antigen distribution. If no specific rabies fluorescence was observed in the impressions, the samples were reported as negative.

All the RABV positive samples used in this study were subjected to antigenic typing to obtain variant information. Antigenic typing was performed on DFA positive brain samples by indirect fluorescent antibody tests using a panel of twenty mAbs against the RABV N protein (anti-N mAbs). RABV variants were determined by the demonstration of established reaction patterns of terrestrial and bat RABVs based on the recognition of N-protein epitopes by a panel of twenty CDC anti-N mAbs as previously established [23]. For MS assay, based on the availability, samples with different levels of antigen distribution, infected with different RABV variants that circulate in the U.S., and from different host species were selected.

### Sample preparation

Purified RABV and lysates from control and RABV infected cells were lysed in 1X NuPAGE LDS sample buffer with reducing agent and boiled at 95°C for 10 min. Infected and uninfected brain tissues (26 samples) were homogenized in 1x PBS (100 mg in 150 μl) followed by boiling with LDS sample buffer at 95°C for 10 min. Proteins were separated in a NuPAGE 4% - 12% Bis-Tris protein gels (proteins are separated under denaturing and reducing conditions) using NuPAGE MES running buffer and stained with Imperial protein stain (ThermoFisher). Either entire lane (12 slices) or specific portions of the gel were excised and processed for in-gel tryptic digestion. The amount of tissue homogenates subjected to electrophoresis corresponds to about 0.6 μg to 2 μg (1 μl– 4 μl) of the samples.

### In-gel tryptic digestion

Gel slices were processed as follows. They were cut into 1 mm x 1 mm cubes followed by three washes of 50% acetonitrile, 10 mM ammonium bicarbonate and dried in a SpeedVac concentrator. Gel pieces were reduced for 60 min at 37°C using 10 mM 1,4-dithiothreitol in 10 mM ammonium bicarbonate and alkylated at room temperature in the dark using 55 mM iodoacetamide in 10 mM ammonium bicarbonate. Gel pieces were again washed three times with 50% acetonitrile, 10 mM ammonium bicarbonate and dried in a SpeedVac concentrator. Samples were rehydrated with sequencing grade modified trypsin (Promega) in 10 mM ammonium bicarbonate and allowed to digest over night at 37°C. The supernatant was collected and gel slices were washed three times with 60% acetonitrile, 10 mM ammonium bicarbonate to extract tryptic peptides. The washes and supernatant were collected, combined, and were dried in a SpeedVac concentrator. About 10%– 25% of tryptic digests obtained from gel slices were subjected for MS analysis.

### Mass spectrometry

Electrospray ionization (ESI) mass spectrometric analysis was performed using a Bruker model maXis ESI-Q-TOF instrument interfaced with a CaptiveSpray ESI spray source (Bruker Daltonics) to perform liquid chromatography–tandem mass spectrometry (LC-MS/MS) using a U3000 RSLCnano HPLC configured for nl/min flows. The Dionex U-3000 RSLCnano nanobore HPLC was configured with a binary nanoflow ultra-high pressure pump and a ternary high pressure microbore pump. The system used a pulled-loop autosampler configured with a 20 μl sample loop. A desalting trap column (0.3 mm x 5 mm, 5 μm C18 PepMap 120 Å, Dionex) was used and the analytical column used was a C18 PepMap (0.075 mm x 250 mm, 2 μm, 120 Å, Dionex). The solvents used were 0.1% formic acid in water (A) and 80% acetonitrile / 0.1% formic acid (B). The gradient was 2%– 55% B in 90 min. The eluent from the analytical column was introduced into the maXis using the Bruker CaptiveSpray source. The source was operated at a spray voltage of 1200 V with a drying gas of nitrogen flowing at 4 liters per min. The capillary temperature was set to 150°C. The mass spectrometer was set to acquire spectra of m/z 50 to 2500. MS/MS data was acquired in an automated fashion using a dynamic precursor ion selection based on the MS scan with precursor ion active exclusion for 60 s after at least 1 spectrum was acquired for each precursor ion. MS data was acquired at a scan speed of 10 Hz and MS/MS data was acquired at a scan speed of 2 Hz– 10 Hz depending on the intensity of the precursor ion. Total cycle time for acquisition of both MS and MS/MS scans was limited to 2.2 s. MS internal calibration was achieved by the use of a lock mass (HP-1222, Agilent Technologies).

### Peptide identification and detection

The collected data was processed by DataAnalysis (Bruker Daltonics) to produce deconvoluted and internally calibrated data and saved as an xml peaklist, which was uploaded to our Proteinscape database (Bruker Daltonics). Proteinscape automatically submitted the peaklist to our in-house MASCOT server for searches against either the Swiss-Prot curated protein Fasta file or a taxonomic filtered data from NCBI’s RefSeq database. The taxonomic filters applied were human and virus.

### Clustal alignment

N protein sequences from RABV variants utilized for this study were obtained from Genbank. The sequences were aligned using Clustal Omega software (https://www.ebi.ac.uk/Tools/msa/clustalo/) to obtain consensus. The peptide fragments sequenced by mass spectrometry were highlighted to demonstrate the conservation or differences observed across various RABV variants.

## Results

### Mass spectrometric analysis of purified RABV

**Fig 1:**
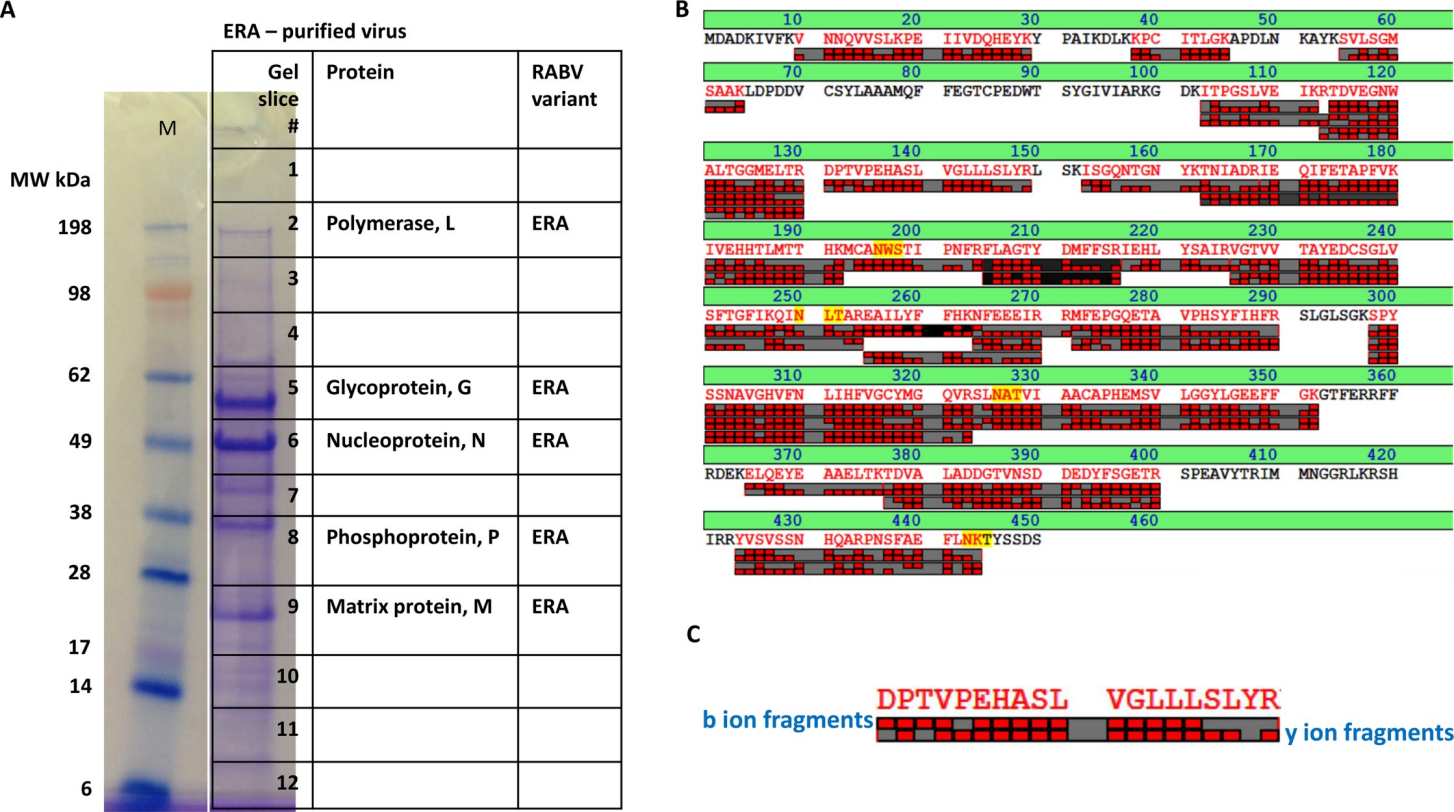
MS analysis of purified RABV ERA virus. (A) RABV ERA virus variant purified from culture supernatants by sucrose density gradient centrifugation was denatured by 1X LDS sample buffer, separated on 4%– 12% Bis-Tris NuPAGE gels and twelve 1-mm slices were analyzed by MS. Molecular weight of protein standards in kilodalton (kDa) is indicated. M–molecular weight marker. The gel slices in which RABV proteins detected are indicated. (B) The peptides identified by MS/MS fragmentation is highlighted in red. The predicted glycosylation sites are highlighted in yellow. (C) One of the MS/MS peptide fragments derived from N protein provided in (B) is enlarged and presented.

To evaluate the potential for mass spectrometric analysis to characterize RABV, purified RABV ERA variant was separated on a protein gel, stained with Imperial protein stain, and subjected to in-gel protein digestion followed by spectrometry (Fig 1A). In the first MS step, based on the experimental peptide mass detected, potential proteins (with theoretical tryptic peptide mass) were identified. In the second MS step, certain precursor peptide ions were subjected to low energy collision and fragmentation into product ions, namely “b” and “y” ions (representing N- and C- terminal fragments respectively) and masses are determined (Fig 1C). As multiple fragments were generated, by comparison of a panel of “b” and “y” ion fragment masses, it was possible to determine amino acid sequence. Based on the peptide mass fingerprint and fragment mass based amino acid sequence of peptides by MS/MS, all five RABV encoded proteins were identified by MS at expected molecular weight positions in the gel slices (Fig 1A). In addition, based on the amino acid sequence determined peptides (denoted in red), N protein present in the sample was clearly identified as RABV ERA variant (Fig 1B). The amino acid sequence coverage by MS/MS for other proteins and the number of unique peptides and percentage of coverage are provided (S1A–S1E Fig). Thus, with sufficient peptide concentrations, fragment mass guided amino acid sequence determination by MS/MS can differentiate and identify the RABV variant present in the sample. MS has the advantage to detect and perform sequence determination for potential variant typing in a single test.

### Detection of RABV proteins in cell lysates

**Fig 2:**
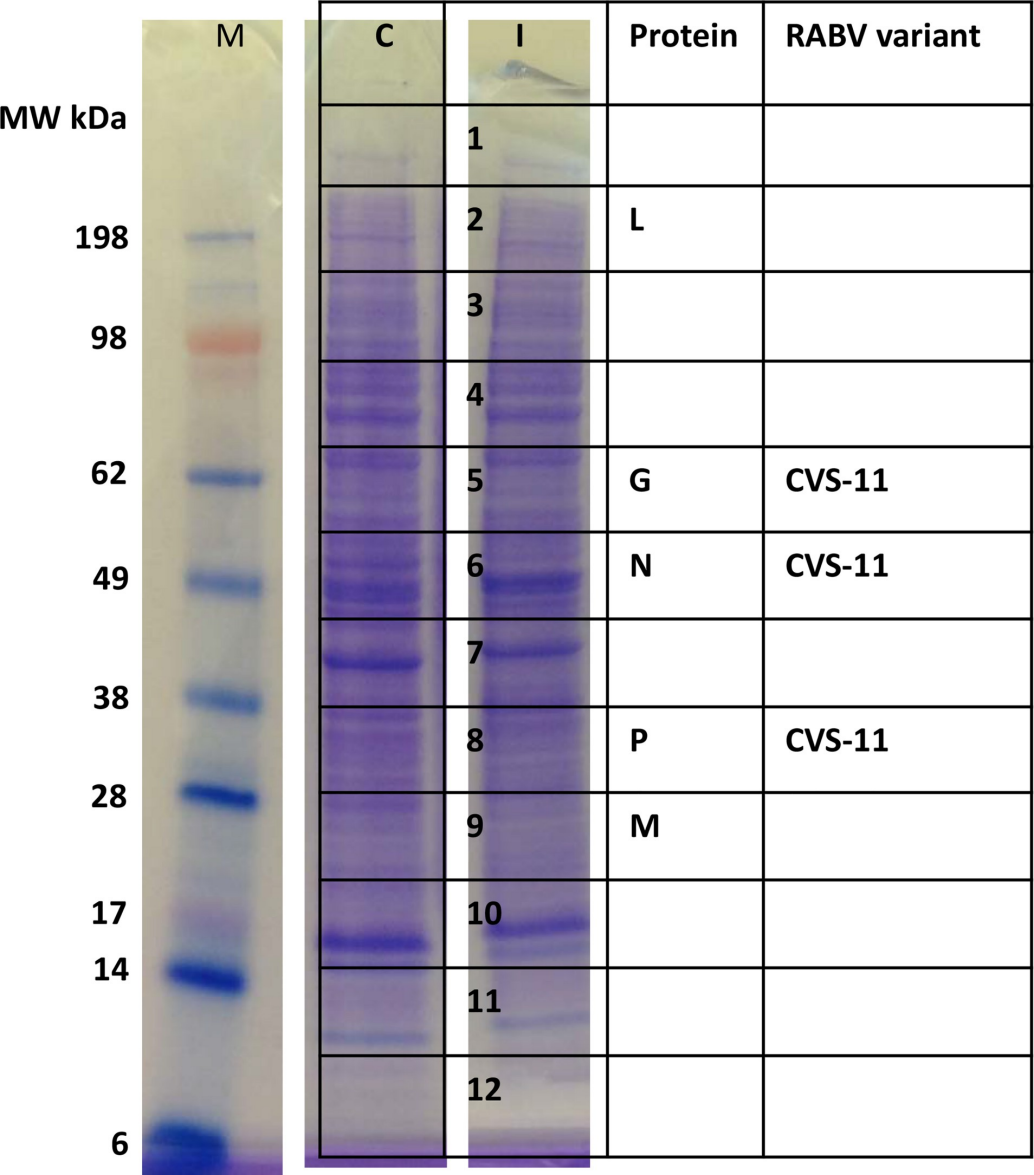
MS analysis of uninfected and RABV CVS-11 infected cell lysates. Mock and RABV CVS-11 infected MNA cells were harvested by centrifugation 24 h post infection. The sample was denatured, separated by gel and analyzed by MS. The slices corresponding to the RABV encoded proteins detected in infected cell lysate lanes by MS are indicated. C and I indicate MNA cells either mock or RABV CVS-11 virus infected cell lysates, respectively. M–protein molecular weight standards in kDa.

To determine the specificity of MS for RABV detection, MNA cell lysates from mock or RABV infected samples were separated on SDS-PAGE, stained by Imperial protein stain, and the entire lane was excised into 12 slices, subjected to in-gel tryptic digestion and analyzed by MS (Fig 2). All five RABV proteins were detected specifically in infected cell lysates at expected molecular weight by MS (as indicated in the gel slices in Fig 2). None of the RABV encoded proteins were detected in any of the 12 slices from the uninfected cell lysate. N protein detection was the highest based on the number of unique peptides and percentage of amino acid sequence coverage, followed by P, M, G and L proteins (S2A–S2E Fig). In addition, based on amino acid sequence determination by MS/MS fragmentation, N, P and G proteins were identified as RABV CVS-11 variant. The number of unique peptides and amino acid sequence coverage are presented in S2F Fig.

### Detection of N protein in infected brain tissues

**Fig 3:**
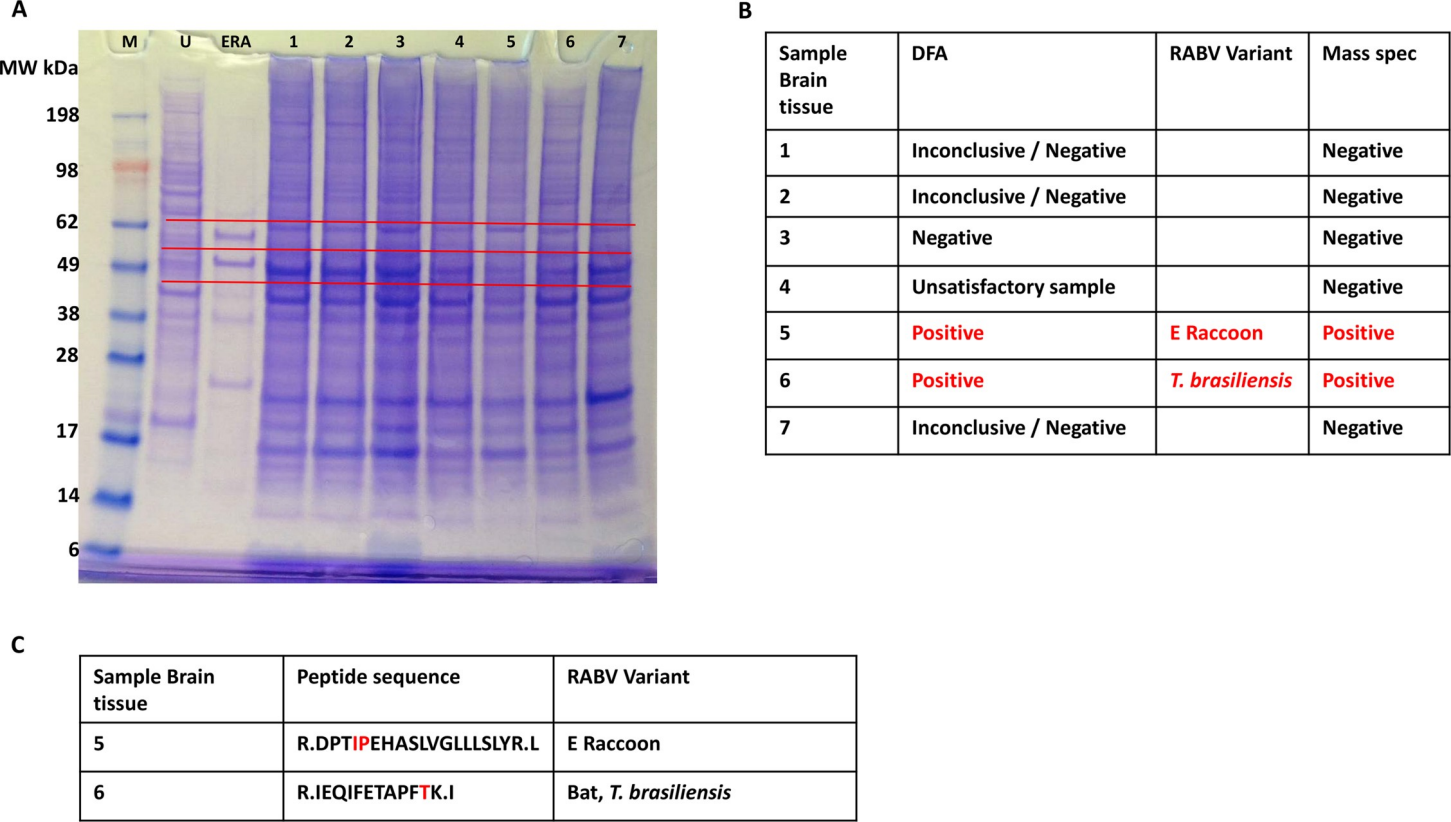
MS analysis of CNS tissues. (A) DFA negative and positive brain tissues homogenates were separated on protein gels and the position of the gel corresponding to the size of N and G proteins based on RABV ERA purified virus were analyzed by MS (red line). M–molecular weight marker in kDa. U–uninfected cell lysate; ERA–purified RABV ERA virus. (B) MS results for the samples analyzed are tabulated. The nature and condition of DFA negative and positive samples, the RABV variant determined by antigenic typing and MS results are provided. (C) The amino acid sequence of peptides determined by MS/MS fragmentation and the unique amino acid used for RABV variant identification are shown in red.

Rabies diagnosis primarily focuses on detection of antigen (N protein) in CNS tissues of animals suspected for rabies. For DFA, touch impressions of brain tissues on slides are tested with anti-N protein specific mAbs or polyclonal Abs. For mass spectrometric detection, tissue homogenates were heat inactivated and separated in a 4%– 12% Bis-Tris protein gel. To limit the number of samples subjected to MS, only the portion of gel corresponding to either N or G proteins (based on the mobility of N and G protein bands in purified RABV ERA) were sliced after staining the gel (Fig 3A). N protein was detected by MS in samples 5 and 6, previously determined positive by DFA. All negative or inconclusive samples were also negative for RABV N protein by MS (Fig 3B). In addition, based on amino acid sequence of unique peptide fragments, both positive samples were correctly identified as either Eastern Raccoon (E. Raccoon) or *Tadarida brasiliensis* (bat) variants of RABV (Fig 3C, described in bioinformatic analysis results section). Although gel slices corresponding to the position of G protein mobility were subjected to MS, the G protein was not detected.

### The limit of N protein detection by MS

**Fig 4:**
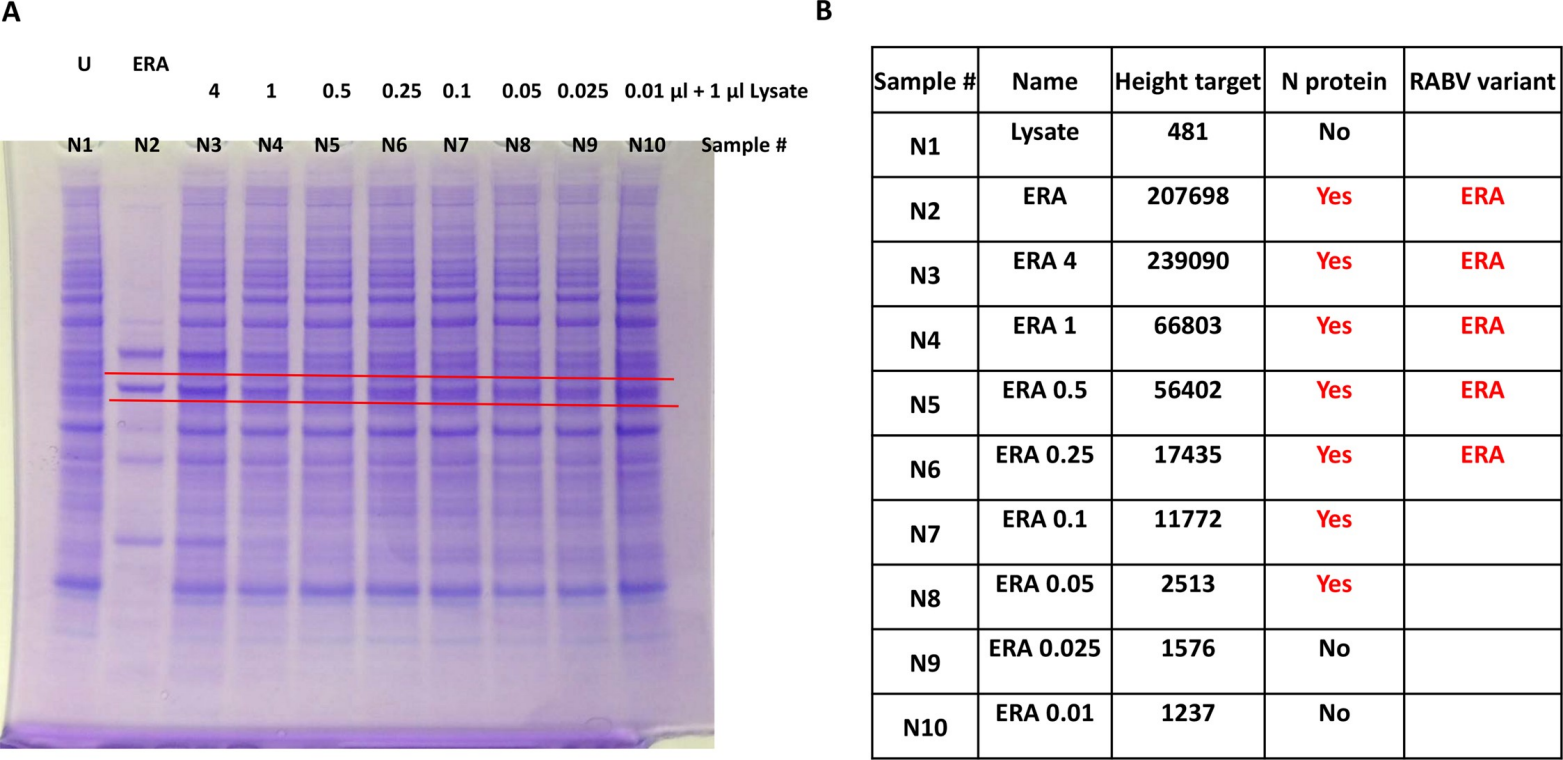
The limit of N protein detection by MS. (A) Different volume of purified RABV ERA virus was added to 1 μl of uninfected cell lysate. The position of the gel corresponding to N protein (marked by red lines) was sliced, in-gel tryptic digested and analyzed by MS. U–uninfected cell lysate, ERA–purified RABV ERA virus. (B) MS results for different gel slices. Height target above 2000 is considered as the cut-off for N protein detection. ERA–denotes to the samples in which N protein variant was identified by MS/MS fragmentation based amino acid sequencing.

The limit of detection of RABV N protein was determined by spiking uninfected cell lysate with purified RABV ERA virus. Different amounts of purified ERA virus at 1.0 X 10^7^ focus forming unit (ffu) / ml (from 4 μl to 0.01 μl) were added to an equal volume of uninfected cell lysate, and were separated by protein gel followed by staining with Imperial protein stain (Fig 4A). The gel position corresponding to N protein was sliced and subjected to MS. N protein was detected in five subsequent dilutions (or equivalent to 5.0 X 10^2^ ffu viral particles) based on at least one peptide above the background values (Fig 4B). At higher dilutions, N protein variant information was not obtained by MS/MS fragmentation analysis probably due to low concentration of peptides. This demonstrates the concentration dependence of MS/MS method for detection of N protein and determination of RABV variants.

### Comparison of MS with DFA

To compare sensitivity of MS for N protein detection with DFA, samples containing different amounts of antigen were tested. Although, DFA is not quantitative, the relative amount of antigen in tissue impressions can be obtained based on the fluorescence intensity and distribution after observation of all microscopic fields. For example, DFA results grade samples from a minimum (1) to maximum (4), with 4+/4+ being maximum fluorescent intensity and distribution, respectively. Generally, anti-N mAbs are titrated to obtain a maximum fluorescent intensity of 4+. Based on the distribution of antigen in microscopic fields, relative levels of N protein in samples are graded from 1+ to 4+. To assess the utility of MS to detect N protein from samples with different RABV variants observed in the U.S. from different species of infected animals and different amounts of antigen, a panel of samples previously analyzed by DFA were tested with the MS assay (S3A and S3B Fig). In general, MS positively identified samples that had higher N protein concentrations as determined by DFA (3+ or 4+ distribution), while samples with lower concentrations (1+ or 2+) were not detected with the instrument used (Table 1). In addition, from the amino acid sequence information obtained by MS/MS fragmentation, E. Raccoon and North Central Skunk (NC Skunk) variants were correctly identified by general database search in two samples (Table 1). Of all the samples tested, G protein was detected only in sample 17 by the MS method.

**Table 1:** The results from MS analysis of CNS tissues.

#	Sample		DFA		Mass spectrometry (N protein)	
	Source	RABV Variant	Result	Distribution	Result	RABV Variant
8	Mouse		Negative		Negative	
9	Dog	NC Skunk	**Positive**	1–2+	Negative	
10	Sheep	E Raccoon	**Positive**	2+	Negative	
11	Dog	E Raccoon	**Positive**	3–4+	**Positive**	**E Raccoon**
12	Dog	NC Skunk	**Positive**	4+	**Positive**	
13	Dog	NC Skunk	**Positive**	4+	**Positive**	**NC Skunk**
14	Cat		Negative		Negative	
15	Dog	E Raccoon	**Positive**	1+	Negative	
16	Horse	SC Skunk	**Positive**	1+	Negative	
17	Fox	AZ Grey Fox	**Positive**	2+	Negative	
18	Cat		Negative		Negative	
19	Dog	E Raccoon	**Positive**	1+	Negative	
20	Raccoon	E Raccoon	**Positive**	1–2+	Negative	
21	Bovine	SC Skunk	**Positive**	3–4+	**Positive**	
22	Bobcat	AZ Grey Fox	**Positive**	4+	**Positive**	
23	Fox	Arctic Fox	**Positive**	4+	**Positive**	
24	Skunk	NC Skunk	**Positive**	4+	**Positive**	
25	Skunk	SC Skunk	**Positive**	4+	**Positive**	
26	Fox	AZ Grey Fox	**Positive**	4+	**Positive**	

Sample–source denotes the CNS tissues of host animals, while RABV variant corresponds to samples from DFA positive CNS tissues determined by antigenic typing. E Raccoon–RABV Raccoon variant observed in Eastern U.S.; NC Skunk and SC Skunk–corresponds to RABV variants circulating in skunks from either North Central or South Central U.S., respectively. AZ gray fox and Arctic fox variants of RABV observed in Arizona and Alaska, respectively.

DFA–Intensity / distribution–corresponds to the semi-quantitative classification of N protein levels based on distribution of anti-N mAb-FITC conjugate on tissue impressions.

Mass spectrometry–Results demonstrate detection of N protein by MS and RABV variant corresponds to identification of RABV variants based on amino acid sequence determination and analysis.

### Bioinformatic analysis of N protein sequence determined by tandem MS/MS

**Fig 5:**
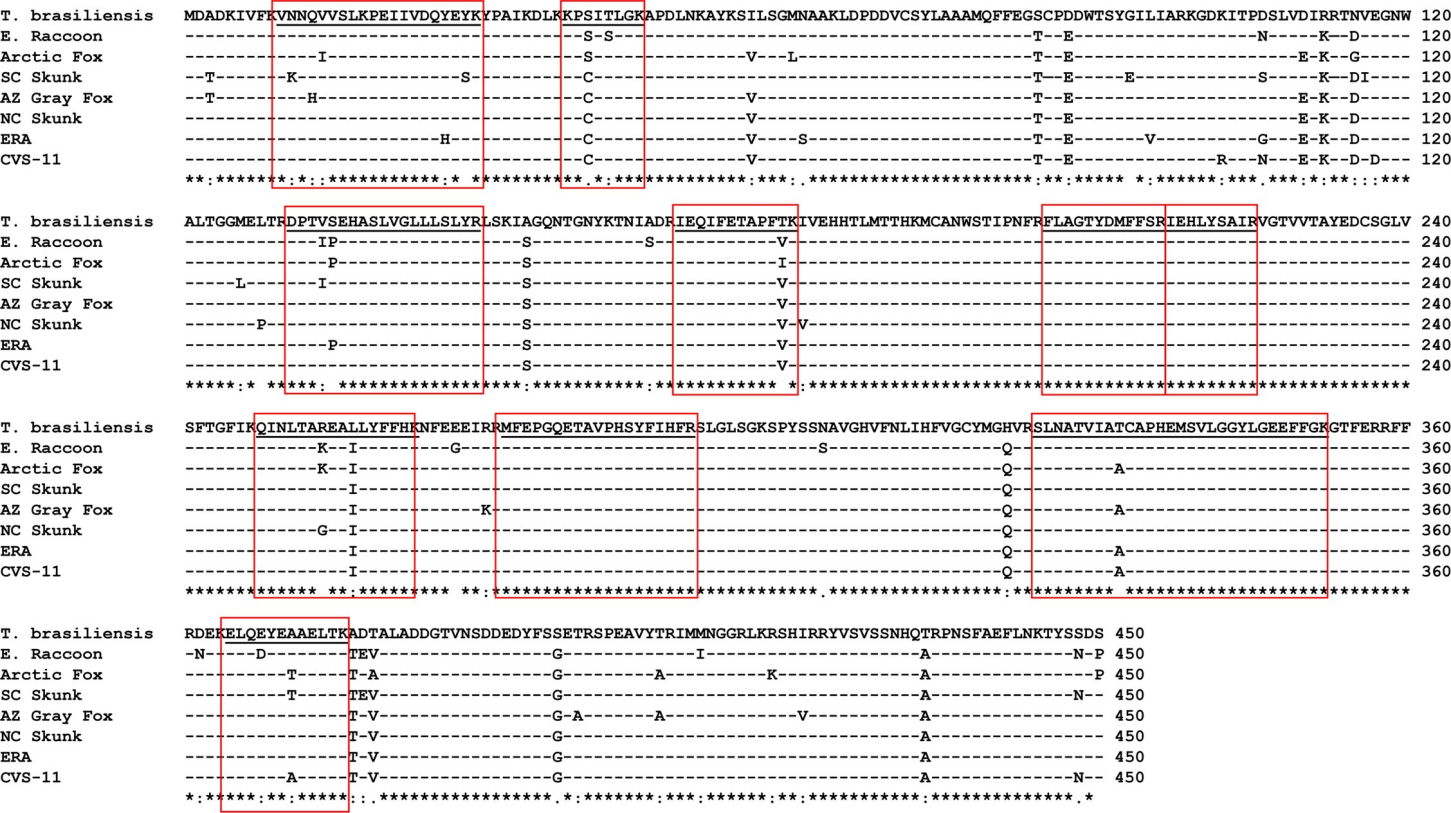
Clustal alignment of N protein amino acid sequences from different RABV variants used in this study. N protein amino acid sequence from representative RABV variants circulating in different hosts are compared using Clustal Omega program. N protein from E Raccoon (GenBank U27221.1), NC Skunk (AF461045.1), SC skunk (ADF80603.1), AZ gray fox (JQ685899.1), Arctic fox (AEV22313.1), *T*. *brasiliensis* (bat, ACN51665.1), CVS-11 (Q8JXF6.1) and ERA (P0DOF3.1) are aligned by Clustal analysis. The most frequently observed peptide fragments in MS are underlined and boxed to demonstrate the conservation or differences in RABV variants.

**Fig 6:**
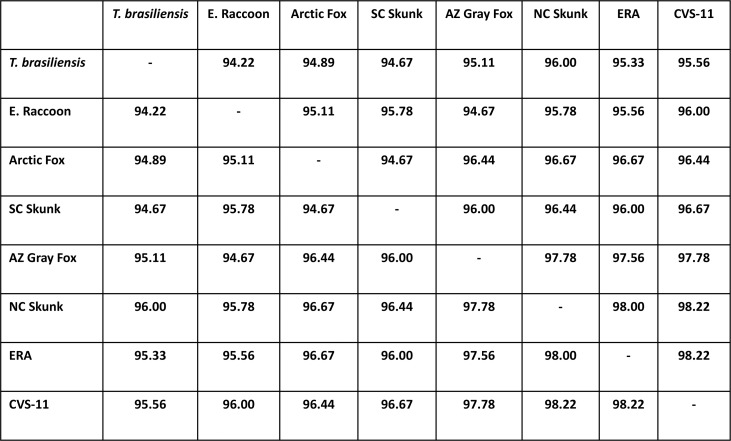
Percentage identity matrix for N protein variants determined by Clustal. The percentage identity of different N protein from RABV variants are tabulated based on the values obtained from Clustal analysis.

**Fig 7:**
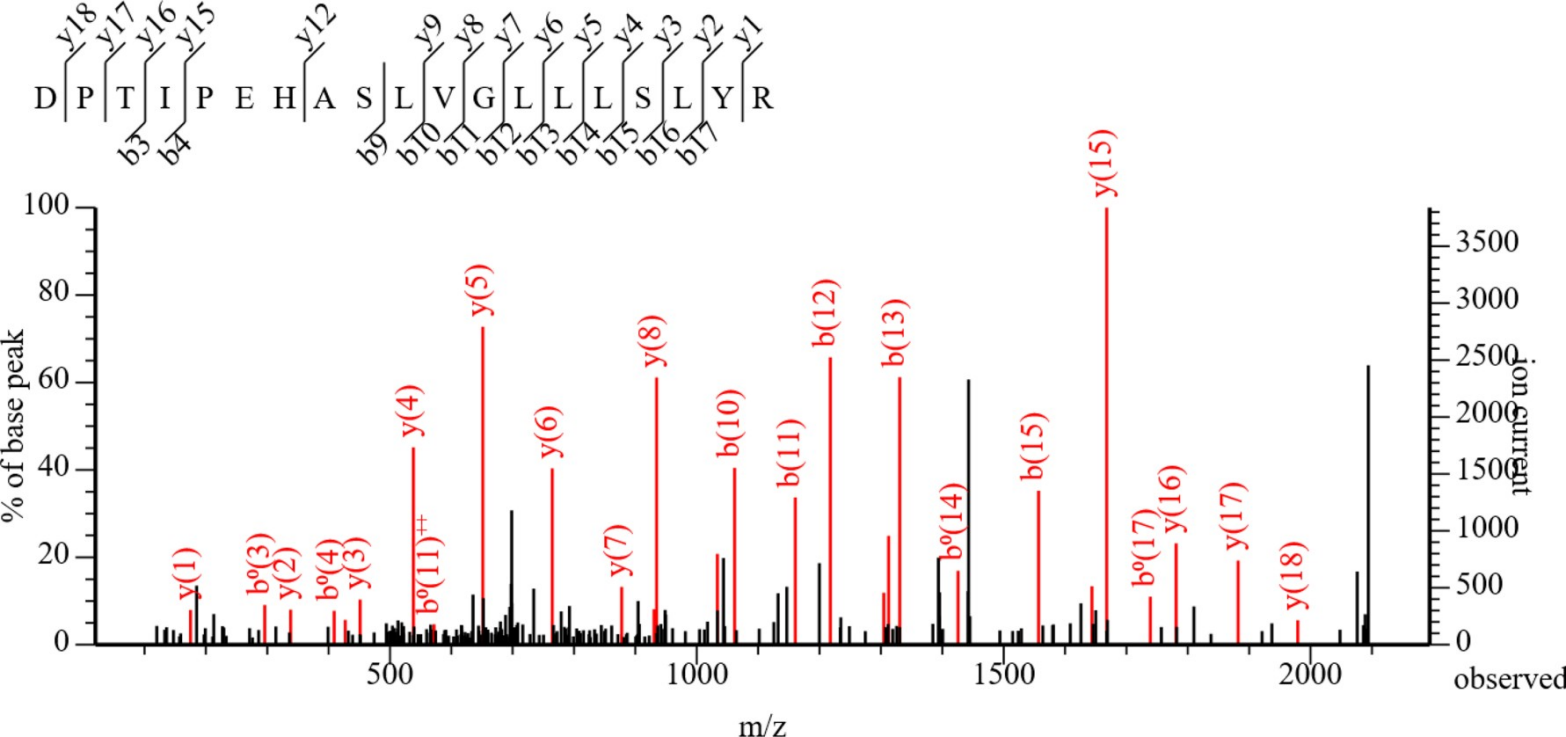
Peptide view. MS/MS fragmentation of peptide DPTIPEHASLVGLLLSYLYR derived from N protein corresponding to E. Raccoon RABV variant. The position and mass of various N- and C- terminal fragments (denoted by b_n_ and y_n_, respectively) are indicated in the graph.

MS/MS fragment mass based amino acid sequence determination has the potential to differentiate RABV variants if peptides containing unique sequences for RABV variants are determined. The list of peptides that contained amino acid residues unique to RABV variants are provided in S1 Spreadsheet. Even though several peptides were identified in both ERA and CVS-11 samples due to higher concentration of N protein in purified virus or experimentally infected samples, only certain peptides could differentiate RABV variants. For ERA, in the peptide VNNQVVSLKPEIIVDQ**H**EYK, H was unique (bold and underlined) compared to other variants (Fig 5). Similarly, in the peptide TDV**D**GNWALTGGMELTR, aspartic acid (D) was unique to CVS-11 N protein sequence. In the subsequent analysis, RABV variants were determined from infected brain tissues corresponding to E. Raccoon, *T*. *brasiliensis* (Fig 3C) and NC Skunk RABV variants (Table 1), while variant information for other positive samples by MS was not obtained by default search results. To improve variant identification, we performed a manual search of peptide fragments based on the Clustal alignment of different N protein sequences for RABV variants used in the study (Fig 5). As the amino acid sequence identity ranged from 94%– 98% (Fig 6), unless peptides containing differences in amino acid sequences are ionized and detected, RABV variant information could not be determined. Identification of RABV E. Raccoon variant in sample 5 was based on amino acid sequence derived from the peptide “DPT**IP**EHASLVGLLLSYLYR” (Fig 7, S4 Fig). In this peptide, amino acid residues in position 4 ("I”) and position 5 (“P”) were unique to E. Raccoon variant. Two additional unique peptides “ELQ**D**YEAAELTK” and “KP**S**I**S**LGK” for E. Raccoon variant were identified in sample 11, in which “D” in amino acid position 4 and “S” in amino acid positions 3 and 5 were present only in this N protein sequence. Interestingly, in one of the peptides derived from NC Skunk variant, “QINLTA**G**EAILYFFHK”, the highlighted “G” in amino acid position 7 is unique and it replaces either a “K” or a “R” in other variants. Since the peptides are generated by proteolytic cleavage with trypsin which cleaves after”K” or “R” residues, this peptide is truncated in other variants leading to this longer peptide only being detected in the NC Skunk variant. Based on the peptide analysis from MS/MS results, South Central (SC) Skunk and Arctic Fox RABV variants were identified. Details of amino acid sequences of peptides utilized for RABV variant identification are provided in S1 Spreadsheet. Only the Arizona (AZ) gray fox variant was not resolved by MS/MS due to the absence of unique peptides. The list of all peptides derived from each sample is provided in a spreadsheet (S2 Spreadsheet). Based on this list, a set of 10 peptides (Fig 5, underlined and boxed) detected in tissue samples and frequency of detection (not considering MS results from purified ERA and infected cell lysate CVS-11) were presented in Table 2. Although, not directly comparable, one of the peptides at the N terminus “VNNQVVSLKPEIIVDQHEYK” encompasses the target for the recently validated LN34 real time RT-PCR [24].

**Table 2:** Based on the amino acid sequence information identified by MS/MS, list of ten peptides and frequency of detection is tabulated.

Peptide	ERA	CVS-11	Sample #											Frequency^$^
			5	6	11	12	13	21	22	23	24	25	26	
K.VNNQVVSLKPEIIVDQHEYK.Y	+	+		+						+	+			3
K.KPCITLGK.A	+	+			+									1
R.DPTVPEHASLVGLLLSLYR.L	+	+	+	+	+	+	+			+	+	+		8
R.IEQIFETAPFVK.I	+	+		+	+		+	+	+	+		+	+	8
R.MFEPGQETAVPHSYFIHFR.S	+	+			+		+	+	+			+		5
R.IEHLYSAIR.V	+	+			+	+	+	+	+	+	+	+		8
R.FLAGTYDMFFSR.I	+	+			+		+		+	+		+		5
K.ELQEYEAAELTK.T	+	+		+	+		+	+	+			+	+	7
R.SLNATVIAACAPHEMSVLGGYLGEEFFGK.G	+	+			+		+				+	+	+	5
K.QINLTAGEAILYFFHK.N	+*						+		+*			+		4

ERA–purified RABV ERA virus, CVS-11 –infected cell lysate and MS positive brain tissue homogenate sample # are provided.

^$^For frequency of detection, peptides detected in ERA or CVS-11 samples are not considered.

*The peptide is truncated in RABV variants other than NC Skunk RABV due to replacement of G with R which adds a trypsin proteolytic cleavage site.

## Discussion

Rabies is a zoonotic disease transmitted by the bite of a lyssavirus infected animal. Due to the lack of specific anti-virals or therapeutics, rabies is considered to have one of the highest case fatality rates for any of the infectious agents after symptom onset [16]. Unfortunately, human rabies diagnosis is not available during the pre-symptomatic phase, which encompasses the time from virus exposure to the establishment of viral replication in the brain [25]. The testing of suspected rabid animals for RABV infection is important to initiate post-exposure prophylaxis for rabies disease prevention in humans who have been bitten. Current rabies diagnostic reagents have been selected by comparison studies to be broadly reactive to highly conserved lyssavirus epitopes, and can be used to detect all of the RABV and lyssaviruses to date albeit at different levels of sensitivity. With new lyssaviruses being discovered, it will be necessary to have reagents that can detect N protein without compromising the specificity of detection. In addition, since the assay depends on the use of antibodies, variations in reagent batches and lots due to purification and conjugation procedures might affect the functionality of the assay. Due to the high expression level of N protein and the characteristic inclusions formed in the cytoplasm of infected cells, microscopy based methods are extremely reliable for rabies diagnosis. Specifically, the DFA is considered the gold standard for rabies diagnostics in animals [16]. Several modifications to detection methods, primary antibodies, and experimental procedures have been developed over the years to detect N protein. Two studies have attempted to determine metabolomics changes as a measure during the initial stages of rabies, however, it still requires additional data to be considered for routine diagnosis [26, 27].

Non-immunomicroscopy based detection of N protein has utilized rapid, point-of-care platforms, including lateral flow assays [19, 21, 28]. These assays rely on two different antibodies that react with N protein, one for antigen capture and the other for detection in diagnostics. Although several products are currently in the market, the results are mixed. The specificity and sensitivity varies with different kits including the limits of detection. The variability in sensitivity raises an important concern with rapid tests and the potential incidence of false negatives [19], that could result in inappropriate recommendations regarding the need for PEP. Although some reagents and protocols demonstrated a high level of concordance with the reference technique DFA [21] and could be utilized for resource limited areas, these assays requires additional confirmatory testing.

Direct detection and sequencing of proteins as a diagnostic for viral infection has not been widely attempted. As each protein has unique amino acid composition and peptide mass fingerprint, it can be used for the specific detection of target proteins. In addition, amino acid sequence of peptides can be determined by MS/MS fragment mass analysis, which further improves the confidence level for protein identifications. In this study, we first demonstrated the ability to detect and obtain RABV variant information for all five RABV encoded proteins by MS in purified RABV particles (Fig 1 and S1 Fig). The specificity of MS was next demonstrated using *in vitro* infected or uninfected cell culture lysates. RABV expressed proteins were detected only from the infected lysate and three of the five proteins had unique sequence information to classify as CVS-11 variant (Fig 2 and S2 Fig). RABV encompasses different variants, primarily based on the adaptation for efficient replication in host reservoir species. These RABV variants have distinct differences in amino acid sequences of encoded proteins as identified in seven major terrestrial wildlife hosts in the U.S. and territories. These include raccoons (E. Raccoon, in East??), Skunks (in NC and SC variants), foxes (AZ Gray, Texas Gray and Arctic [Alaska]), mongoose (in Puerto Rico), and at least 14 RABV variants associated with different bat species which are ubiquitous in the U.S. In this study, we demonstrate detection of N protein in clinical specimens from major RABV variants observed in the U.S.

The two major observations obtained from this study are, the ability (1) to detect N protein from crude tissue preparation without any amplification or N protein specific reagents and (2) to obtain amino acid sequence information for further confirmation and identification of RABV variants. Samples that were classified as 3–4+ antigen distribution by DFA, were predominantly positive by MS, while samples with lower N protein content were not sensitive enough for detection using the Bruker maXis QTOF instrument. Of the 18 DFA positive samples tested, N protein was detected by MS in 11 samples (61% sensitivity). As majority of samples have higher levels of antigen, the MS assay described in this study would have higher levels of sensitivity in actual field samples. In addition to confirmation of N protein in tissue samples, all but one of the RABV variants were determined based on amino acid sequence information. The current N protein detection method by immunomicroscopy requires two separate tests for RABV variant determination. Once RABV N proteins are confirmed by DFA, a panel of 20 anti-N mAbs that bind differentially to RABV variants are tested by IFA. Based on the pattern of reactivity of these anti-N mAbs and comparison with established terrestrial and bat variant patterns, the RABV variant is identified. In spite of high level of sequence identity exhibited by N protein, amino acid sequencing of unique peptides were able to differentiate RABV variants (Fig 5). This is particularly significant as only changes in nucleotides that results in amino acid change (non-synonymous substitutions) are detected in MS, compared to both synonymous and non-synonymous substitutions obtained by DNA sequencing. Based on MS/MS results, high-resolution peptide sequence analysis that can differentiate E. Raccoon, *T*. *brasiliensis*, NC Skunk, SC Skunk and Arctic Fox RABV variants were identified (Fig 5, Fig 7, S1 Spreadsheet). Thus, our results demonstrate for the first time N protein detection and sequencing by MS/MS directly from RABV infected animal CNS tissue samples. Although we also attempted to detect G protein in infected tissues, except for one sample, it was not detected by MS. The detection of N and not G protein, further substantiates the current diagnostic methods focus on N protein detection due to higher level of expression followed by RABV infection.

The MS method was successful in identifying several major RABV variants circulating in the U.S. and from several different host animal species. The results from this study provide information on peptides derived from the N protein that are readily ionizable and detectable by MS. Peptides conserved across all RABV variants or containing unique sequences for differentiation of variants could be employed for either species- or variant- specific MS-based assays (S1 Spreadsheet). Furthermore the development of a PRM (parallel reaction monitoring) type target based assay, would allow for multiplexing since the MS instrumentation can be programmed to scan for dozens of potential targets and for the selective quantification of proteins in samples [29, 30]. While this study focused on RABV variants that circulate in the U.S., MS analysis of RABV variants or non-rabies lyssaviruses observed in other countries will be helpful to generate a peptide database for species and variant specific detections.

In this study, we have explored MS as an alternative method and obtained preliminary data on the feasibility for rabies N protein detection. We will further explore the possibility to detect N protein directly from brain suspensions, instead of separating by protein gels using PRM methods to improve the sensitivity of detection compared to DFA. Although, MS-based assays may not be cost effective at present, it could be an additional antigen/protein detection method relying not on antibodies and microscopy at reference laboratories. With advancement in MS and increased use in clinical laboratories, it offers a next generation technology platform for exploring rabies antigen detection. Another major advantage of recent “-omics” technologies are identification of multiple pathogens or pathogen discovery from a single assay. As the MS-based assay is unbiased and does not need specific reagents, it can be employed for pathogen identification for samples with unknown etiology (negative for RABV, but exhibiting neurological symptoms) by “characterization of proteomics” similar to “metagenomics” approach.

## Supporting information

S1 Fig MS results of purified RABV ERA virus. RABV ERA protein sequences and the position of identified peptides sequenced by MS/MS fragment analysis are denoted for P protein (A), M protein (B), G protein (C) and L protein (D). (E) The number of unique peptides identified and total percent coverage of amino acid sequences in all five RABV encoded proteins. All four proteins were identified as RABV ERA variant based on MS/MS results. The amino acid residues in red demonstrate the peptides for which sequence was deduced and the yellow highlighted residues corresponds to the predicted glycosylation sites.
(TIF)

S2 Fig MS results of RABV CVS-11 virus infected MNA cell lysates. RABV CVS-11 protein sequences and the position of identified peptides sequenced by tandem MS/MS are denoted for N protein (A), P protein (B), M protein (C), G protein (D) and L protein (E). (F) The number of unique peptides identified and total percent coverage of amino acid sequences in all five RABV encoded proteins. N, P and G proteins corresponded to CVS-11, while M and L proteins corresponded to PM1503/AV01 RABV variants based on the MS/MS results. The amino acid residues in red demonstrate the peptides for which sequence was deduced and the yellow highlighted residues corresponds to the predicted glycosylation sites.
(TIF)

S3 Fig Protein gel electrophoresis. (A) and (B) Imperial protein stained gels of different CNS tissue samples analyzed by MS. M–molecular weight market and sizes, ERA–purified ERA virus and sample numbers are provided on top of each lane. The position of gel slices are marked by red lines.
(TIF)

S4 Fig Peptide fragmentation. The molecular mass of b_n_ and y_n_ fragment ions described in Fig 7. Based on the differences in mass between different fragments, the potential amino acid residues are predicted. With sequential analysis of b_n_ and y_n_ ion masses, amino acid sequence information of peptide is deduced.
(TIF)

S1 Spreadsheet Ten peptides and the corresponding unique or conserved sequences, used for RABV variant determinations from the analysis are listed. (XLSX)

S2 Spreadsheet All N protein specific peptides identified by MS/MS in different samples are listed. (XLSX)
