# Supplementary figures and images for: Novel mass spectrometry based detection and identification of variants of rabies virus nucleoprotein in infected brain tissues
Source: PLoS Negl Trop Dis. 2018 Dec 14;12(12):e0006984. doi: 10.1371/journal.pntd.0006984 (PMC6310296; doi:10.1371/journal.pntd.0006984)

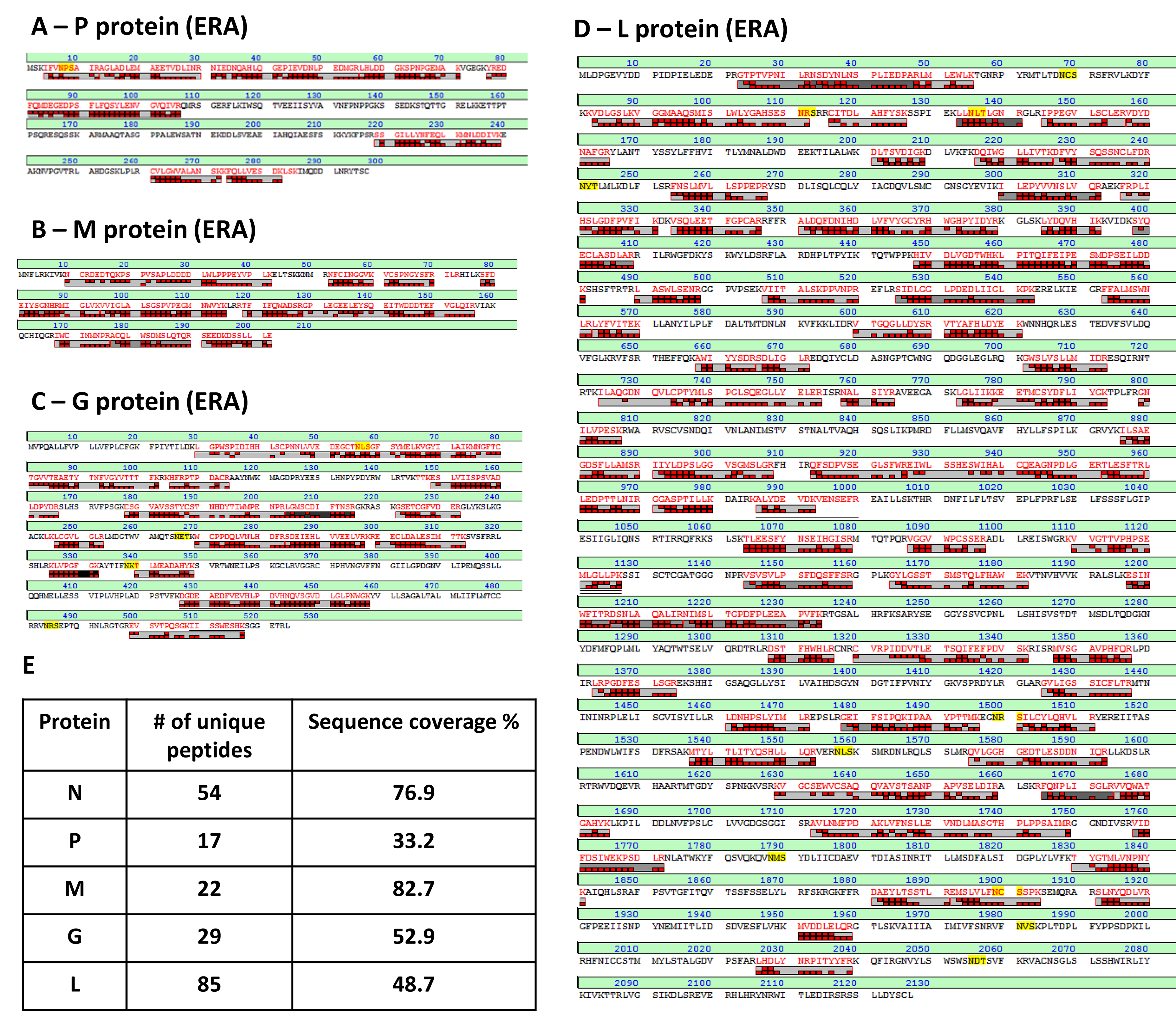

Supplement: S1 Fig — RABV ERA protein sequences and the position of identified peptides sequenced by MS/MS fragment analysis are denoted for P protein (A), M protein (B), G protein (C) and L protein (D). (E) The number of unique peptides identified and total percent coverage of amino acid sequences in all five RABV encoded proteins. All four proteins were identified as RABV ERA variant based on MS/MS results. The amino acid residues in red demonstrate the peptides for which sequence was deduced and the yellow highlighted residues corresponds to the predicted glycosylation sites. (TIF) [file pntd.0006984.s001.tif]

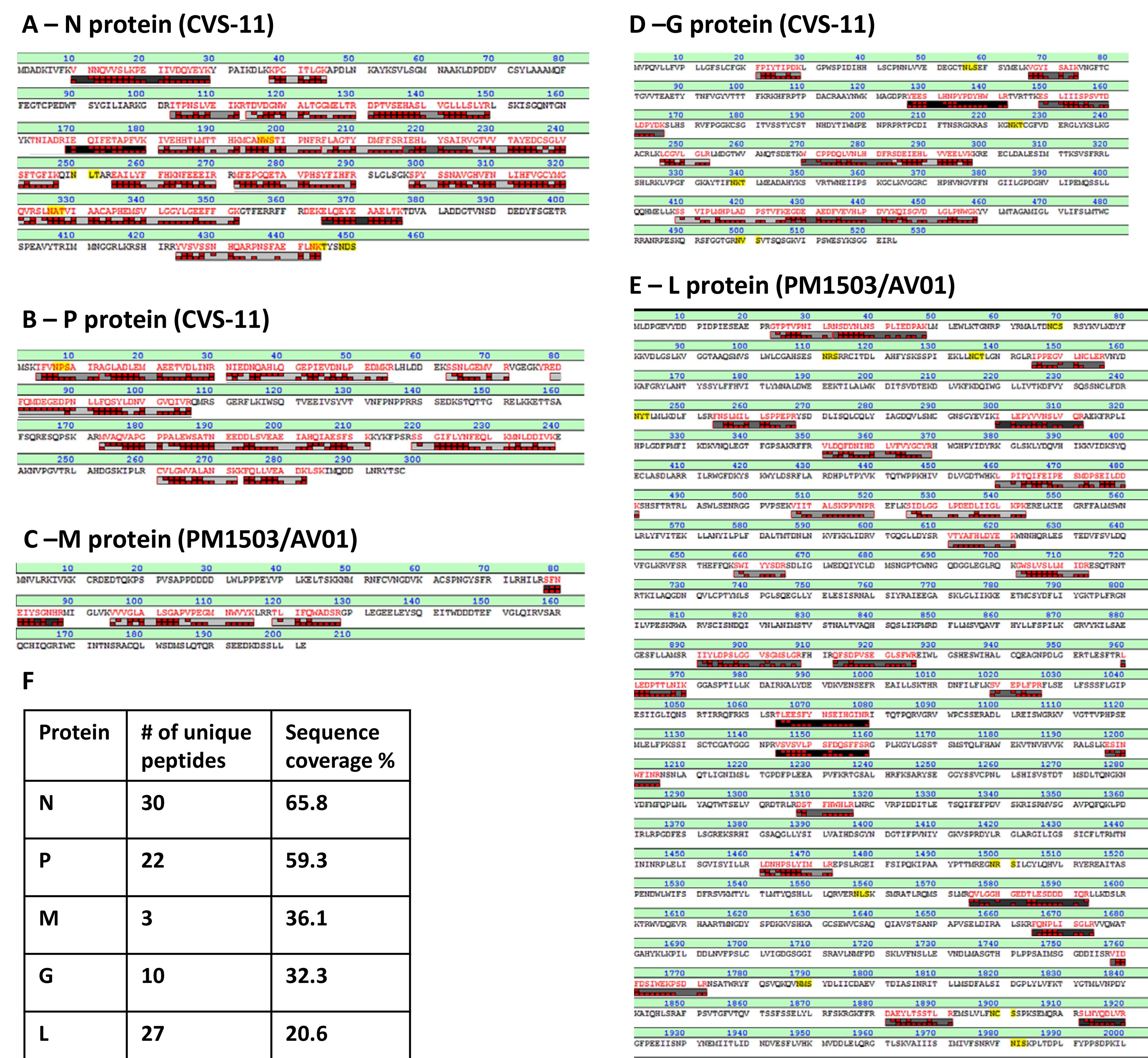

Supplement: S2 Fig — RABV CVS-11 protein sequences and the position of identified peptides sequenced by tandem MS/MS are denoted for N protein (A), P protein (B), M protein (C), G protein (D) and L protein (E). (F) The number of unique peptides identified and total percent coverage of amino acid sequences in all five RABV encoded proteins. N, P and G proteins corresponded to CVS-11, while M and L proteins corresponded to PM1503/AV01 RABV variants based on the MS/MS results. The amino acid residues in red demonstrate the peptides for which sequence was deduced and the yellow highlighted residues corresponds to the predicted glycosylation sites. (TIF) [file pntd.0006984.s002.tif]

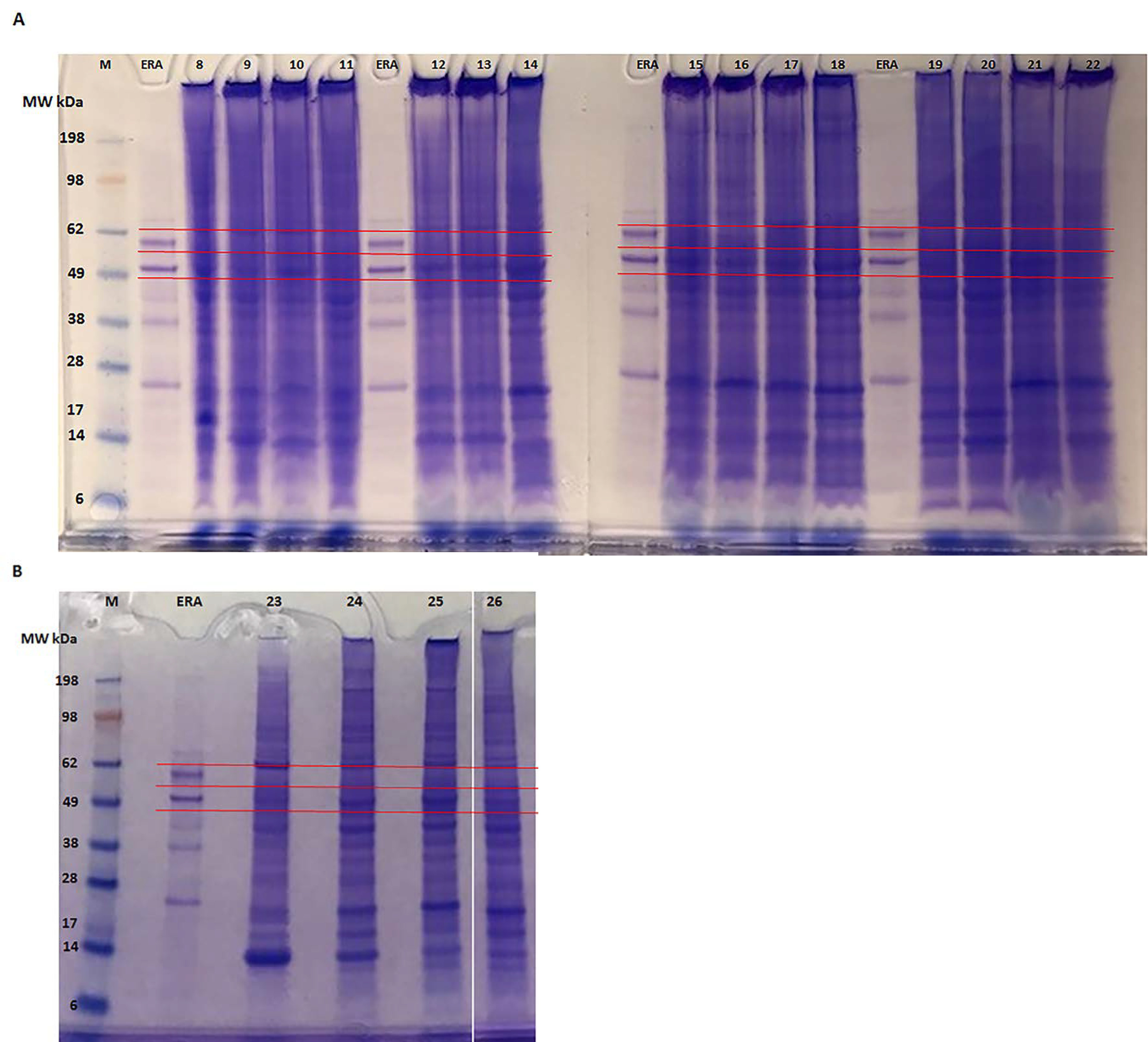

Supplement: S3 Fig — (A) and (B) Imperial protein stained gels of different CNS tissue samples analyzed by MS. M–molecular weight market and sizes, ERA–purified ERA virus and sample numbers are provided on top of each lane. The position of gel slices are marked by red lines. (TIF) [file pntd.0006984.s003.tif]

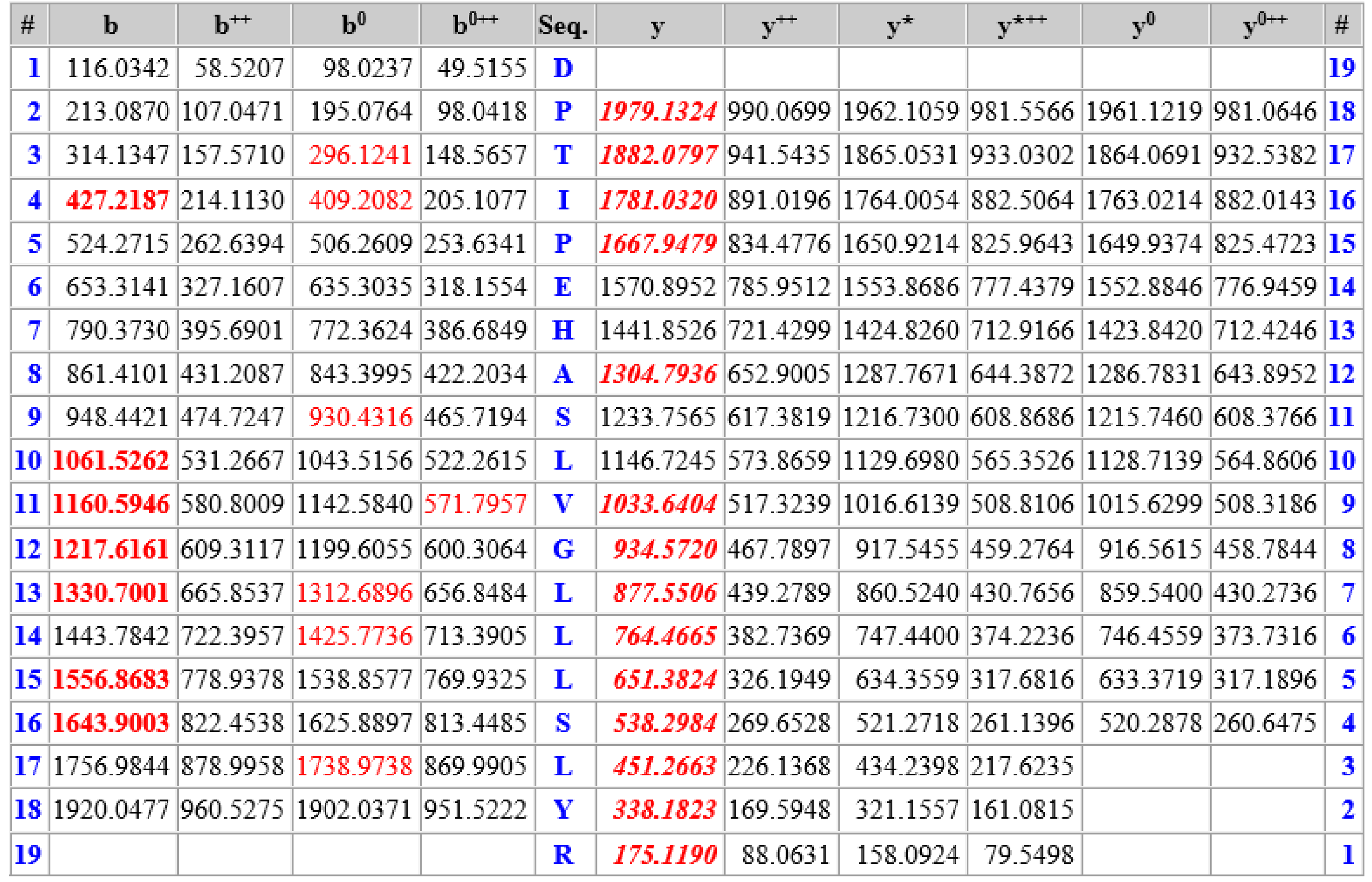

Supplement: S4 Fig — The molecular mass of bn and yn fragment ions described in Fig 7. Based on the differences in mass between different fragments, the potential amino acid residues are predicted. With sequential analysis of bn and yn ion masses, amino acid sequence information of peptide is deduced. (TIF) [file pntd.0006984.s004.tif]
